# ﻿Two new species of genus *Leucoagaricus* and *Leucocoprinus* (Agaricaceae, Agaricales) from China

**DOI:** 10.3897/mycokeys.125.160410

**Published:** 2025-11-14

**Authors:** An-Qi Zhang, Ri Jin, Xue-chao Zhang, Entaj Tarafder, Ji-Ze Xu

**Affiliations:** 1 Agricultural College, Yanbian University, Yanbian 133000, China Agricultural College, JiLin Agricultural Science and Technology College Jilin China; 2 Agricultural College, JiLin Agricultural Science and Technology College, Jilin 132000, China Yanbian University Jilin China; 3 Jilin Provincial Horticultural and Special Products Management Station, Changchun 130000, China Jilin Provincial Horticultural and Special Products Management Station Changchun China; 4 Center for Yunnan Plateau Biological Resources Protection and Utilization & Yunnan International Joint Laboratory of Fungal Sustainable Utilization in South and Southeast Asia, College of Biology and Food Engineering, Qujing Normal University, Qujing 655099, China Qujing Normal University Qujing China

**Keywords:** Lepiotoid fungi, molecular phylogeny, morphology, novel taxa, taxonomy

## Abstract

In this study, two new species, *Leucoagaricus bailangshanensis* and *Leucocoprinus ferrugineus*, were discovered. Phylogenetic analyses based on ITS, nrLSU, *rpb*2, and *tef*1-*α*sequences confirm that these taxa represent distinct lineages within well-supported monophyletic groups. *Leucoagaricus bailangshanensis* is characterized by a yellow-orange center on the pileus, a margin bearing floccules, and relatively long sterigmata, which distinguish it from closely related species. *Leucocoprinus ferrugineus* can be recognized by its mahogany-red pileus center, a white annulus with a tomato-red margin that turns brown when bruised, and ellipsoid basidiospores. Detailed morphological descriptions, along with color photographs, illustrations, and a phylogenetic tree, are provided to clarify the taxonomic positions and relationships of these two new species.

## ﻿Introduction

Locquin first classified *Leucoagaricus* as a subgenus under *Leucocoprinus* Pat (Locquin 1943). In 1948, Singer elevated *Leucoagaricus* togenus with a formal Latin diagnosis (Singer 1948). Candusso and Lanzoni (1990) later reclassified the Leucoagaricus into two subgenera viz., subgenus Sericeomyces and subgenus Leucoagaricusm, and six sections viz., sect. Cystidiosi Migl. & Testoni, sect. Intermedii (Bon) Consiglio & Contu, sect. Leucoagaricus Locq. ex Singer, sect. Piloselli Singer, sect. Pseudopiloselli Migl. & Coppola, and sect. Rubrotincti Singer (Bon 1981). *Leucoagaricus* is characterized by the small to medium-sized, thin or fleshy basidiomata; pileus surfaces range from radially fibrillose, floccose, squamulose to fibrillose-scaly or rarely granulose; entire or very short striated margins; central, equal to bulbous stipe that have membranous, sometimes fugaciousannulus; thin-walled and smooth basidiospores generally lack well-defined germ pores; and the pileipellis is either a trichoderm or a cutis of repent and radially arranged hyphae lacking clamp connections absent; spore print white, sordid cream, or cream-orange; and metachromatic in Cresyl Blue (Singer 1986; Vellinga 2001). The majority of *Leucoagaricus* species have been documented from temperate regions in North America and Europe, whereas only a limited number have been reported from tropical areas (Liang et al. 2010; Ishaq et al. 2025).

Patouillard segregated *Leucocoprinus* Pat from *Lepiota*, but lacked an original circumscription. Locquin (1943) later provided a comprehensive systematic description of *Leucocoprinus* and subdivided it into three subgenera: *Leucocoprinus*, *Leucoagaricus*, Hiatula (Fr.) Mont., and *Leucobolbitius* J.E. Lange ex Locq. The genus *Leucocoprinus* is primarily distributed in tropical and subtropical regions (Vellinga 2001, 2004, 2011; Ge et al. 2015; Liang et al. 2010; Vellinga et al. 2010). *Leucocoprinus* is a highly diverse genus, with the majority of its species occurring in tropical regions (Vellinga 2004). In recent years, numerous new species have been described from these areas (Ge et al. 2015; Liang et al. 2010; Malysheva et al. 2013; Li et al. 2025). In 2001, Vellinga defined the diagnostic characteristics of *Leucocoprinus* as follows: basidiospores are metachromatic, clamp connections are absent, pseudoparaphyses are present around the basidia, and the pileus has a plicate margin (Vellinga 2001). Subsequently, most researchers have consistently used these characteristics to define species within *Leucoagaricus* (Justo 2020, 2021).

The *Leucoagaricus* and *Leucocoprinus* exhibit a cosmopolitan distribution in Europe (Candusso and Lanzoni 1990), Pakistan (Ashraf et al. 2023), and Laos (Sysouphanthong 2018). Currently, *Leucoagaricus* comprises more than 130 species and *Leucocoprinus* 250 species globally, based on the legitimate names in the MycoBank (https://www.mycobank.org) and the Species Fungorum database (www.speciesfungorum.org; accessed September 24, 2025). To date, a total of 12 *Leucoagaricus* and 33 *Leucocoprinus* species have been reported in China, such as: *Leucoagaricus atrosquamulosus* Z. W. Ge & Zhu L. Yang (Yang 2017), *Leucoagaricus exannulatus* Singer (Dong 2013), *Leucoagaricus lateritiopurpureus* E. F. Malysheva, T. Yu. Svetasheva & E. M. Bulakh (Fan 2022), *Leucoagaricus bulbosus* R. L. Zhao & J. X. Li, *Leucoagaricus xantholepis* R. L. Zhao & J. X. Li, *Leucoagaricus testaceumbonatus*R. L. Zhao & J. X. Li, *Leucoagaricus luteocanus* R. L. Zhao & J. X. Li, *Leucoagaricus cinereibisporus*R. L. Zhao & J. X. Li, *Leucoagaricus centrobrunneolus* R. L. Zhao & J. X. Li, *Leucoagaricus subcandidus* R. L. Zhao & J. X. Li, *Leucoagaricus subnivalis* R. L. Zhao & J. X. Li (Li et al. 2025), *Leucocoprinus brunneoruber*J. M. Zhang & X. T. Zhu (Zhu 2024), *Leucocoprinus coerulescens* J. F. Liang, Zhu L. Yang & J. Xu (Liang et al. 2010), *Leucocoprinus shixingensis* (Z.S. Bi & T.H. Li) Kun L. Yang, Jia Y. Lin & Zhu L. Yang (Yang et al 2024), and *Leucocoprinus subcretaceus* Bon (Chu 2014).

In the current taxonomic revisions, sous-genus *Leucoagaricus* and sous-genus *Sericeomyces* are still placed within *Leucoagaricus*; the remaining related taxa have been reclassified into *Leucocoprinus* (Yang et al. 2024). At present, the phylogenetic relationships between *Leucocoprinus* and *Leucoagaricus* remain unresolved. Previous studies have primarily focused on ITS and nrLSU analyses, resulting in incomplete molecular data; therefore, this study employs ITS, nrLSU, rpb2, and *tef*1-*α* sequences. In this study, two new species of *Leucoagaricus* and *Leucocoprinus* from China are described based on both morphological and molecular data. The abbreviations for the generic names referenced in this study are as follows: ***L.*** = *Lepiota*, ***La.*** = *Leucoagaricus*, and ***Lc.*** = *Leucocoprinus*.

## ﻿Materials and methods

### ﻿Collection of specimens

The study area is located in the northeastern part of China, where we found two new species. The species of *Leucoagaricus* found in Liaoning Province, specifically in Huludao City, Bailangshan National Nature Reserve, grows on soil and is solitary in deciduous broadleaf forests in August. The species of *Leucocoprinus* found in Nei Mongol, specifically in Hinggan League City, Arxan National Forest Park, is scattered on the soil in a coniferous-broadleaf mixed forest in September. Fresh basidiocarps were photographed using a Canon 80D digital single-lens reflex (DSLR) camera, with concurrent documentation of habitat characteristics (Rathnayaka et al. 2025). The color characteristics of the basidiocarps were documented and coded following the methodology of Kornerup and Wanscher (1978). All specimens were dried overnight in an electric blast drying oven at 45 °C (Hu et al. 2022) and then deposited in the Herbarium of Mycology at Jilin Agricultural Science and Technology University (**HMJU**).

### ﻿Morphological observation

Detailed macro-morphological descriptions of the collected specimens were accomplished from the fresh specimens. Microstructural features, including basidiospores, basidia, cheilocystidia, pleurocystidia, and pileipellis elements, were observed using the protocols described by Largent (1986). The dried specimen sections were processed through fixation with 3% potassium hydroxide (KOH), 1% Congo red, and Melzer’s reagent, and subsequently observed. [n, p, m] indicates: The data represent n basidiospores measured from p samples within m specimens. Basidiospore dimensions are presented in the format ‘(a–) b–av–c(–d)’, where the range ‘b–c’ encompasses at least 90% of measured values. Extreme values a and d are indicated in parentheses, with ‘av’ representing the average. Q denotes the length-to-width ratio of spores, and Qm represents the mean value of Q for all basidiospores. Electron Microscope (SEM) images of basidiospores were obtained from dried, free-hand sections of lamellae, directly mounted on a double-sided adhesive tape pasted onto a metallic specimen stub, and then scanned at different magnifications in high-vacuum mode (Tarafder et al. 2022, 2025). This work was conducted using a Zeiss EVO 18 electron microscope.

### ﻿DNA extraction, PCR amplification, and sequencing

Total genomic DNA was extracted using the EZup Column Fungi Genomic DNA Purification Kit (Sangon Biotech Co., Ltd., Shanghai, China) according to the manufacturer’s protocol. During PCR amplification, primers ITS1F and ITS4 were employed to amplify the ITS rDNA region (Gardes 1993; White 1990), primers LR0R and LR5 were used to obtain nrLSU sequences (Vilgalys and Hester 1990), primers *rpb*2-6F and *rpb*2-7cR were utilized to target the *rpb*2 gene (Hofstetter et al. 2007; Matheny 2005), and primers TEF1-983F and TEF1-1567R were applied to amplify the *tef*1-α region (Qu et al. 2023). The PCR cycling protocol comprised: initial denaturation at 95 °C for 5 min, followed by 28 cycles (ITS) or 32 cycles (nrLSU) or 30 cycles *(rpb*2, *tef*1-α) of denaturation at 94 °C for 30 s (ITS, *tef*1-α) or 94 °C for 40 s (nrLSU) or 94 °C for 50 s (*rpb*2), annealing at 54 °C for 30 s (ITS) or 52 °C for 45 s (nrLSU) or 59 °C for 1 min (*rpb*2) or 49 °C for 35 s (*tef*1-α), and extension at 72 °C for 30 s (ITS, *tef*1-α) or 72 °C for 40 s (nrLSU) or 72 °C for 1 min (*rpb*2), with a final extension step at 72 °C for 5 min (ITS) or 72 °C for 4 min (nrLSU, *tef*1-α) or 72 °C for 10 min (*rpb*2). The PCR products detected by 1% agarose gel electrophoresis using the JY 600 electrophoresis (Beijing JUNYI Electrophoresis Co., Ltd., Beijing, China) apparatus were sent to BGI Co., Ltd. (Beijing, China) for sequencing.

### ﻿Sequence acquisition and dataset preparation

Use the BLAST algorithm to compare the sequences obtained in this study against the GenBank nucleotide database (ITS, nrLSU, *rpb*2, and *tef*1-*α*). Accession numbers were obtained from GenBank upon submission of nucleotide sequences to the NCBI database. In the phylogenetic analysis, 16 new sequences were generated from two specimens. Integrate the newly generated sequences with existing data from previous phylogenetic studies (Misra et al. 2011; Yang et al. 2024), using *Lepiota grangei* (Eyre) Kühner and *Lepiota felina* (Pers.) P. Karst. as the outgroup (Sarawi 2025) to construct a combined data matrix.

### ﻿Sequence alignment and phylogenetic analyses

To confirm the taxonomic positions of the new species, ITS and nrLSU, *rpb*2, and *tef*1-*α*, sequences were combined and analyzed with Bayesian inference (BI) and maximum likelihood (ML) methods. Sequence alignment was performed using MAFFT v7.0, (Katoh and Standley 2013), and low-quality regions were trimmed with MEGA v7.0 (Kumar et al. 2016) and then combined by Phylosuite v1.2.3 (Zhang et al. 2020; Xiang et al. 2023). The BI analysis was conducted using MrBayes v3.2.7a (Ronquist et al. 2012) in Phylosuite v1.2.3, which employs a Markov chain Monte Carlo (MCMC) algorithm. Nucleotide substitution models were determined by ModelFinder v 2.2.0 (Kalyaanamoorthy et al. 2017). Four Markov chains were run simultaneously for 1,000,000 generations, with the trees sampled every 1000 generations. A 75% majority rule consensus tree was computed after excluding the first 25% trees as “burn-in”. Bayesian inference posterior probability (BIPP) was determined from the remaining trees. The ML analysis was performed using IQ-Tree v2.2.0 (Nguyen et al. 2015) in Phylosuite v1.2.3, with the best model selected for each locus according to ModelFinder. The model selection was performed using ModelFinder under the Bayesian Information Criterion (BI) (Kalyaanamoorthy et al. 2017), with the best model being GTR+F+I+G4.

The phylogenetic position of the new species was inferred using Maximum Likelihood (ML) and Bayesian Inference (BI) methods. The phylogenetic analysis was performed using IQ-TREE for Maximum Likelihood (Minh et al. 2020) and Markov Chain Monte Carlo (MCMC) methods, with MrBayes 3.2.2 (Ronquist et al. 2012). Bootstrap values ≥ 70%in Maximum Likelihood (ML) analysis and Bayesian posterior probabilities (PP) ≥ 0.90 are considered to provide significant support for phylogenetic nodes. The sequences used in this study are listed in Table 1.

**Table 1.** Specimens used in molecular phylogenetic studies, along with their corresponding GenBank accession numbers.

Species	Voucher	GenBank accession number				References
		ITS	nrLSU	*rpb2*	*tef1-α*	
*Leucoagaricus bulbosus*	ZRL20232333	PP816091	PP816160	PQ305429	PQ356770	(Li et al. 2025)
*Leucoagaricus bulbosus*	ZRL20232692	PP816093	PP816161	PQ305428	PQ356769	(Li et al. 2025)
*Leucoagaricus bulbosus*	ZRL20234728	PP816092	PP816159	PQ305427	PQ876894	(Li et al. 2025)
*Leucoagaricus centrobrunneolus*	ZRL20234390	PP816185	PP816158	PQ305434	PQ356765	(Li et al. 2025)
*Leucoagaricus centrobrunneolus*	ZRL20234710	PP816115	PP816157	PQ305433	PQ356766	(Li et al. 2025)
*Leucoagaricus centrobrunneolus*	ZRL20234413	PP816116	PP816155	PQ305435	PQ356768	(Li et al. 2025)
*Leucoagaricus centrobrunneolus*	ZRL20234707	PP816117	PP816156	PQ305432	PQ356767	(Li et al. 2025)
*Leucoagaricus cinereibisporus*	ZRL20232133	PP816136	PP816162	PQ305418	PQ356773	(Li et al. 2025)
*Leucoagaricus cinereibisporus*	ZRL20232134	PP816137	PP816163	PQ305419	PQ356771	(Li et al. 2025)
*Leucoagaricus cinereibisporus*	ZRL20235342	PP816138	PP816164	PQ305417	PQ356772	(Li et al. 2025)
*Leucoagaricus subcandidus*	ZRL20234776	PP816127	PP816153	PQ305431	PQ876890	(Li et al. 2025)
*Leucoagaricus subcandidus*	ZRL20236826	PP816128	PP816154	PQ305430	PQ876891	(Li et al. 2025)
***Leucoagaricus bailangshanensis***	**HMJU9098**	**PV593136**	**PV595846**	**PV645742**	**PV645746**	**This study**
***Leucoagaricus bailangshanensis***	**HMJU9304**	**PV593137**	**PV595847**	**PV645743**	**PV645747**	**This study**
*Leucoagaricus subnivalis*	ZRL20232605	PP816099	PQ618975	PQ634984	PQ876898	(Li et al. 2025)
*Leucoagaricus subnivalis*	ZRL20232733	PP816100	PQ618977	PQ634985	PQ876899	(Li et al. 2025)
*Leucoagaricus testaceumbonatus*	ZRL20234316	PP816187	PP816149	PQ305424	PQ356775	(Li et al. 2025)
*Leucoagaricus testaceumbonatus*	ZRL20234323	PP816139	PP816148	PQ305422	PQ356774	(Li et al. 2025)
*Leucoagaricus testaceumbonatus*	ZRL20234352	PP816140	PP816150	PQ305423	PQ356776	(Li et al. 2025)
*Leucoagaricus xantholepis*	ZRL20232065	PP816102	PP816152	PQ305425	PQ876886	(Li et al. 2025)
*Leucoagaricus xantholepis*	ZRL20232816	PP816104	PP816151	PQ305426	PQ876887	(Li et al. 2025)
*Leucocoprinus beijingensis*	ZRL20232057	PP816072	PP816167	PQ305414	PQ305440	(Li et al. 2025)
*Leucocoprinus beijingensis*	ZRL20235282	PP816074	PP816169	PQ305416	PQ305437	(Li et al. 2025)
*Leucocoprinus beijingensis*	ZRL20233848	PP816071	PP816171	PQ305412	PQ305438	(Li et al. 2025)
*Leucocoprinus beijingensis*	ZRL20232533	PP816075	PP816170	PQ305411	PQ305439	(Li et al. 2025)
*Leucocoprinus beijingensis*	ZRL20233576	PP816076	PP816172	PQ305413	PQ305436	(Li et al. 2025)
*Leucocoprinus brebissonii*	ECV1784	AF482859	AY176446	HM488851	HM488931	(Vellinga. 2004)
*Leucocoprinus cepistipes*	ZD16070520	MN523275	HM488779	HM488844	—	(Vellinga. 2011)
*Leucocoprinus cepistipes*	HTBM0275	PQ321873	PQ319799	—	—	(Yang et al. 2024)
*Leucocoprinus cepistipes*	HTBM1402	PQ321883	PQ319809		PQ329242	(Yang et al. 2024)
*Leucocoprinus digitatocystis*	ZRL20232285	PP816069	PP816174	PQ305406	PQ356674	(Li et al. 2025)
*Leucocoprinus digitatocystis*	ZRL20235328	PP816070	PP816173	PQ305407	PQ356673	(Li et al. 2025)
***Leucocoprinus ferrugineus***	**HMJU745**	**PV593135**	**PV595844**	**PV645741**	**PV645745**	**This study**
***Leucocoprinus ferrugineus***	**HMJU898**	**PV595849**	**PV595845**	**PV645740**	**PV645744**	**This study**
*Leucocoprinus leucothites*	HMAS88854	EU416308	EU416309	JN993680	—	(Liang et al. 2010)
*Leucocoprinus licmophorus*	HTBM0578	PQ321872	PQ319798	—	—	(Yang et al. 2024)
*Leucocoprinus licmophorus*	HTBM0689	PQ321879	PQ319805	PQ329233	PQ329244	(Yang et al. 2024)
*Leucocoprinus lilacinogranulosus*	10440 4	KM083044	—	—	—	(Tian. 2014)
*Leucocoprinus purpurascens*	ZRL20234956	PP816079	PP816175	PQ305410	PQ876874	(Li et al. 2025)
*Leucocoprinus purpurascens*	ZRL20236943	PP816080	PP816176	PQ305409	PQ876875	(Li et al. 2025)
*Leucocoprinus* sp.	ZRL20233912	PP816067	PQ618991	—	—	(Li et al. 2025)
*Leucocoprinus* sp.	ZRL20234966	PP816131	PQ619004.1	—	—	(Li et al. 2025)
*Leucocoprinus tangerinus*	HTBM0679	PQ321878	PQ319804	—	PQ329243	(Yang et al. 2024)
*Leucocoprinus tangerinus*	HTBM0836	PQ321867	PQ319794	—	—	(Yang et al. 2024)
*Leucocoprinus tangerinus*	HTBM0958	PQ321868	PQ319795	—	—	(Yang et al. 2024)
*Leucocoprinus tangerinus*	HTBM1779	PQ321870	PQ319796	—	—	(Yang et al. 2024)
*Lepiota grangei*	KaiR1768	PP594624	PP594638	PP841166	—	(Sarawi et al. 2025)
*Lepiota felina*	SeSa7	PP594535	PP594651	PP841258	—	(Sarawi et al. 2025)

## ﻿Results

### ﻿Phylogenetic analyses

**Figure 1.**
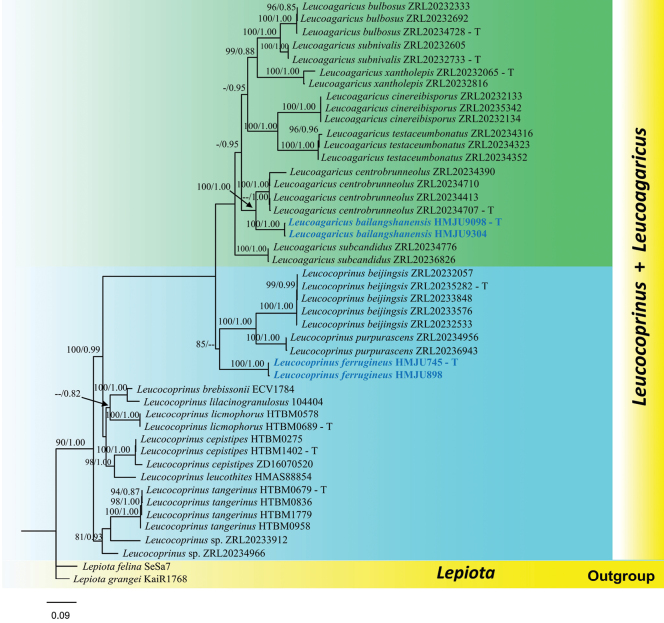
Phylogram generated from maximum likelihood analysis based on ITS, LSU, *rpb*2, and *tef*1-α sequences data representing *Leucoagaricus* and *Leucocoprinus* species. Related sequences are obtained following Li et al. (2025) and GenBank. The tree topology of the ML analysis is similar to the Bayesian analysis. Bootstrap values for ML equal to or greater than 70% and clade credibility values greater than 0.90 (rounded to 2 decimal places) from BYPP analysis are labeled on the nodes. A newly generated sequence is in blue bold, and the type specimen sequences are represented in T.

The final dataset included 16 newly generated sequences and 153 sequences retrieved from the GenBank database. Two sequences of *Lepiota grangei* and *L. felina* from GenBank were selected as outgroups. The ML and BI analyses yielded the same topologies, with maximum likelihood bootstrap percentage (MLBP) values on the left and Bayesian posterior probability (PP) values on the right; thus, only the ML tree is presented (Fig. 1). In the resulting phylogeny, the Chinese specimens clustered into two distinct clades with moderate to strong statistical support, indicating that they represent two undescribed species. Within *Leucoagaricus*, the newly generated ITS, nrLSU, *rpb*2, and *tef*1-α sequences of *La. bailangshanensis* (HMJU 9098) formed a sister relationship to *La. centrobrunneolus* (ZRL20234707) with 100% ML and 1.00 BYPP statistical support (Fig. 1). Within *Leucocoprinus*, the ITS, nrLSU, *rpb2*, and *tef1-α* sequences of *Lc. ferrugineus* (HMJU 745) grouped as sisters to *Leucocoprinus beijingensis* R. L. Zhao & J. X. Li (ZRL20235282) and *Leucocoprinus purpurascens* T. Guo & Z. W. Ge (ZRL20234956), though this relationship was supported only by moderate ML values of 85% and unsupported in the Bayesian analysis.

### ﻿Taxonomy

#### ﻿ Leucoagaricus bailangshanensis

Taxon classificationFungiAgaricalesAgaricaceae

J.Z. Xu sp. nov.

B8B0CBFD-01DD-563B-833D-F928E496A5AC

Fungal Names: FN 573018

Fig. 2

##### Diagnosis.

*Leucoagaricus bailangshanensis* differs from *La. centrobrunneolus* by pileus margin with floccules, longer sterigmata (3.5 µm), and distinctive ITS, nrLSU, *rpb*2, and *tef*1-*α* sequences and position in the phylogram.

**Figure 2.**
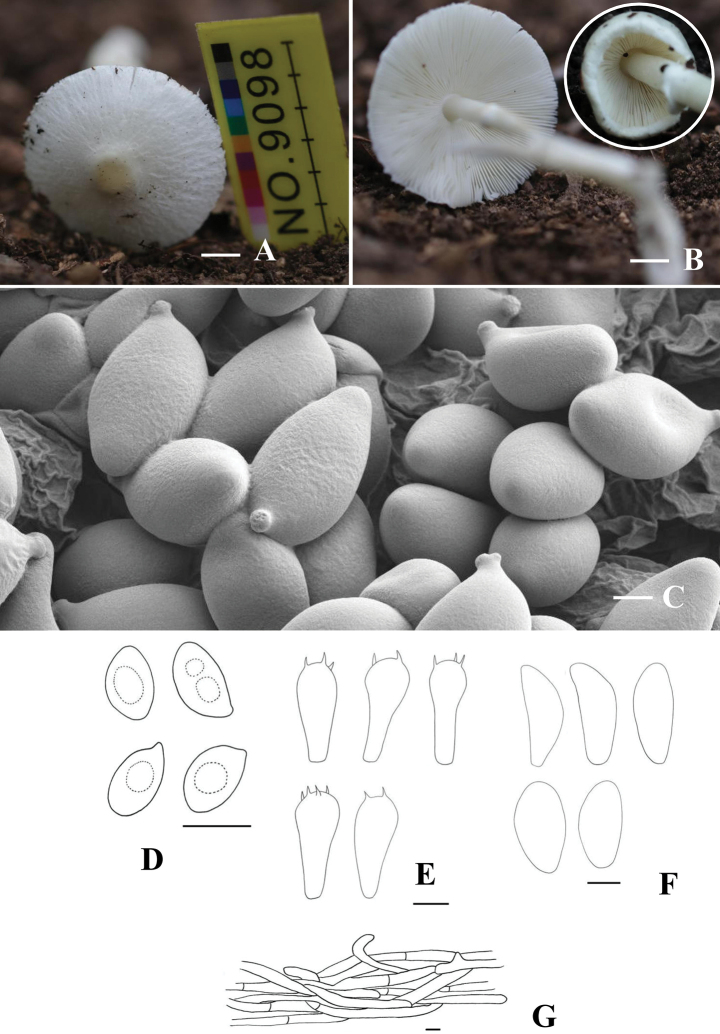
*Leucoagaricus bailangshanensis* (HMJU 9098, holotype). A, B. Basidiocarps; C. SEM images of basidiospores; D. Basidiospores; E. Basidia; F. Cheilocystidia; G. Pileipellis. Scale bar: 2 cm (A, B); 2 µm (C); 5 µm (D, E); 10 µm (F, G).

##### Holotype.

**China** • Liaoning Province, Huludao City, Bailangshan National Nature Reserve, on soil, 8 August 2023, J. Z. Xu (HMJU 9098, holotype).

##### Etymology.

The epithet “*bailangshanensis*” refers to the location ‘Bailang shan of Liaoning Province, where the holotype was collected.

##### Description.

***Pileus*** 33–48 mm diam, ranging from plano-convex to convex, pale grayish-white to snow-white (28B1-28A1), with an irregular margin exhibiting remnants of the partial veil, surface arid, bearing imbricate squamules and radiating white fibrillose vestiges; umbo slightly obtuse, vinaceous yellow (4B4) centrally positioned, becoming radially fissured from the pileus center upon maturation. Context white, non-discoloring when bruised, thin. ***Lamellae*** free, cream-white (1A2) to white (1A1), crowded, less than 1 mm in width, with 1–3 tiers of lamellulae; edge entire. ***Stipe*** 41–54 × 3–5 mm, birch bark (6B2), hollow, covering white fibrillose. ***Annulus*** superior and single-layered white (1A1) on the upper stipe. ***Odor*** not distinctive. ***Spore*** print white.

***Basidiospores*** [40/4/2] (3.6–)4.7–5.6–6.4(–6.9) × (2.8–)3.1–3.9–4.5(–4.8) µm, Q=1.2–1.8, Qm=1.40, broadly ellipsoid to slightly elongated, without a germ pore, transparent in 3% KOH, dextrinoid, with 1 or 2 guttules. ***Basidia*** (12–)13–14.8–17(–17.7) × (5.0–)6.5–7.3–8.1(–8.7) µm, 2–4 spored, broadly clavate, hyaline in KOH; sterigmata up to 3.5 µm long. ***Cheilocystidia*** (23.1–)23.4–29.4–37.4(–39.4) × (7.3–)9.0–11.4–13.7(–15.0) μm, subfusiform to fusiform, smooth and hyaline KOH. ***Pleurocystidia*** absent. ***Lamellatrama*** regular, made up of parallel to subparallel, 2.6–8.1 μm wide hyphae in KOH. ***Pileipellis*** a cutis of repent, subcylindrical, radially arranged, occasionally branched, trichodermal, hyphae 3.6–14.2 wide. ***Clamp*** connections absent.

##### Habitat.

Solitary on soil in deciduous broadleaf forests.

##### Known distribution.

Known only from north-eastern China.

##### Additional material examined.

**China** • Liaoning Province, Huludao City, Bailangshan National Nature Reserve, on soil, 8 August 2023, J.Z. Xu (HMJU 9304); **China** • Jilin Province, Jilin City, Zuojia Nature Reserve on soil, 22 September 2023, J.Z. Xu (HMJU 9842).

##### Notes.

The species is characterized by a pileus with a vinaceous, yellow umbo and white squamules radially arranged over a white background, crowded lamellae, a pileus margin with floccules, and cheilocystidia subfusiform to fusiform; and ITS, nrLSU, *rpb*2, *tef*1-α sequence analyses, the present species clusters with members of the La. centrobrunneolus, signifying its position. Regarding overall morphology, the present specimen is quite similar to *La. centrobrunneolus*, *La. lateritiopurpureus*, *La. Goossensiae* Heinem, *Leucoagaricus fuligineus* Pegler, *Leucoagaricus griseus* Heinem, and *La. luteocanus*. However, *La. centrobrunneolus* has a much smaller pileus (20 mm) with its surface covered without floccules; smaller sterigmata (1.5 µm) (Li et al. 2025). The pileus of *La. lateritiopurpureus* (10–20 mm) is covered with pink-brownish, brightly orange-brown, or terracotta scales, and much bigger basidia (20–25 × 7–10 µm) (Malysheva et al. 2013). *La. goossensiae* and *La. bailangshanensis* differ in that the pileus is reddish-brown, smoother, and with radiating grooves; and the stipe is longer (Heinemann 1973). *Leucoagaricus fuligineus* has a pileus the center of which is grayish-brown; the hyphae of the pileipellis are grayish-brown; basidia are bigger; and spores are concave (Pegler 1977). *Leucoagaricus griseus* has a pileus, the center of which is dark gray, with much larger basidia and lanceolate cheilocystidia (Heinemann 1979). *Leucoagaricus luteocanus* has a pileus the center of which is light brown, with radial light-brown to pale-yellow squamulose or fibrillose; the stipe has uneven brown coloration.

#### ﻿ Leucocoprinus ferrugineus

Taxon classificationFungiAgaricalesAgaricaceae

J.Z. Xu sp. nov.

1151C4BA-1D5C-5CA9-B2CC-0F37AE06F658

Fungal Names: FN 573019

Fig. 3

##### Diagnosis.

*Leucocoprinus ferrugineus* differs from *Lc. purpurascens* by the absence of cheilocystidia, basidiospores amygdaliform, smaller basidia (14.7– 21.5 × 6.4–9.5 µm) (Guo et al. 2023) and distinctive ITS, nrLSU, *rpb*2, and *tef*1-α sequences and position in the phylogram.

**Figure 3.**
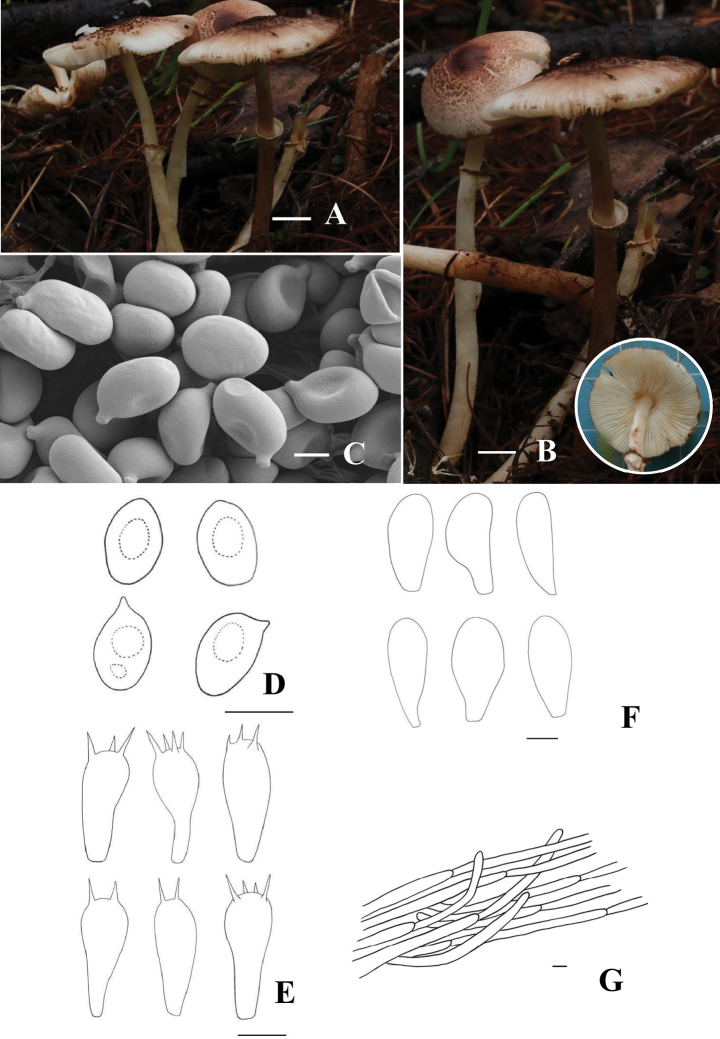
*Leucocoprinus ferrugineus* (HMJU 745, holotype). A, B. Basidiocarps; C. SEM images of basidiospores; D. Basidiospores; E. Basidia; F. Cheilocystidia; G. Pileipellis. Scale bar: 2 cm (A, B); 2 µm (C); 5 µm (D, E); 10 µm (F, G).

##### Holotype.

**China** • Nei Mongol, Hinggan League City, Arxan National Forest Park, on soil, 1 September 2020, J. Z. Xu (HMJU 745, holotype).

##### Etymology.

The species epithet “*ferrugineus*” is derived from the Latin word “ferruginous”, referring to the ferruginous red pileus at the center of the taxon.

##### Description.

***Pileus*** 12–35 mm diam, convex-lens, mahogany-red (8E7) blunt umbo at center and small mahogany-red (8E7) squamules in white base color; margin uneven oxide-red (8E8). ***Context*** white, discoloring to oxide red (8E8) when bruised, thick. ***Lamellae*** free, white with densely radiating tomato-red (8C8) spots, less than 1 mm in width with 1–3 tiers of lamellulae; edge entire. ***Stipe*** 35–50 × 2–3 mm, cylindrical with a swollen base, white in the lower part, gradually deepening to fox (8D7). ***Annulus*** at the upper middle portion of the stipe, white, with a mahogany-red (8E7) margin.

***Basidiospores*** [40/4/2] (5.9–)6.8–7.5–8.5(–8.9) × (4.1–)4.3–4.9–5.6(–6.2) µm, Q=1.4–1.9, Qm=1.54, broadly ellipsoid to slightly elongated, without germpore, transparent in 3% KOH, dextrinoid, with 1 or 2 guttules. ***Basidia*** (13.1–)14.7–18.1–21.5(–21.8) × (6.3–)6.4–7.9–9.5(–10.4) µm, 2–4 spored, broadly clavate; sterigmata up to 2.5 µm long, hyaline in KOH. ***Cheilocystidia*** (28.0–)34.0–44.7–59.8(–71.2) × (11.1–)12.2–16.5–21.3(–28.2) µm, capitate to subcapitate, smooth and hyaline in KOH. ***Pleurocystidia*** absent. ***Lamellatrama*** regular, made up of parallel to subparallel, 3.26–12.53 μm wide, hyaline in KOH. ***Pileipellis*** a cutis of repent, subcylindrical, radially arranged, occasionally branched, trichodermal, seen hyphae 3.0–13.5 μm wide, transparent in 3% KOH. ***Clamp*** connections absent.

##### Habitat.

Solitary on the soil in coniferous-broadleaf mixed forest.

##### Known distribution.

Known only from north-eastern China.

##### Additional material examined.

Nei Mongol • Hinggan League City, Arxan National Forest Park, on soil, 1 September 2020, J. Z. Xu. (HMJU 898)

##### Notes.

The species is characterized mainly by mahogany-colored squamules at the center, stipe fox-brown in median to lower portions, deepening in color toward the base, annulus white with a mahogany-colored margin, context turns oxide red upon injury, and the spores are ellipsoid and ITS, nrLSU, *rpb*2, *tef*1-α sequence analyses, the present species cluster with members of the *Lc. beijingensis* and *Lc. purpurascens* signifies its position. Among morphologically related species, the present specimen is somewhat similar to species such as *Lc. purpurascens*, *Leucocoprinus lahorensis* Asif, Saba & Vellinga, *Leucocoprinus antillarum* Justo, Bizzi, Angelini, *Leucocoprinus brunneocanus*, *Leucocoprinus brunneus*. Among morphologically related taxa, *Lc. purpurascens* differs in lacking cheilocystidia and pileipellis hyphae, which are typically yellowish-brown (Guo et al. 2023). *Leucocoprinus antillarum* has larger spores, cheilocystidia that are subfusiform, and basidiomata that are uniformly white. *Leucocoprinus brunneocanus* Asif, Saba & Vellinga has gray squamules of pileus; context un-discoloring when bruised; and cheilocystidia sometimes constricted or curved (Asif 2024). *Leucocoprinus brunneus* differs by having a thicker stipe; cheilocystidia are smaller and clavate.

## ﻿Discussion

Phylogenetic analyses based on the combined ITS, nrLSU, *rpb*2, and *tef*1-α dataset show a monophyletic clade of *Leucoagaricus* and *Leucocoprinus* taxa, including *Lepiota* as outgroup (Fig. 1). Detailed morphological features, along with phylogenetic studies, support the *La. bailangshanensis* and *Lc. ferrugineus* as new species. *Leucoagaricus bailangshanensis* shows a close phylogenetic affinity with *La. centrobrunneolus*, originally described from China (ITS: GenBank PP816117); however, *La. centrobrunneolus* is characterized by a much smaller pileus, larger basidia, and smaller sterigmata. *Leucocoprinus ferrugineus* exhibits the closest phylogenetic affinity to *Lc. beijingensis* (ITS: GenBank PP816074; Li et al. 2025), and *Lc. purpurascens* described from China (ITS: GenBank OM987458; Yang et al. 2024). *Leucocoprinus beijingensis* differs from *Lc. ferrugineus* by possessing pyriform cheilocystidia and a context that does not change color when damaged (Zhang et al. 2020). *Leucocoprinis purpurascens* differs in lacking cheilocystidia and possessing yellowish-brown hyphae in the pileipellis.

To date, a total of 12 species of *Leucoagaricus* and 33 species of *Leucocoprinus* have been reported in China. Based on the observations of characteristic keys and literature, we prepared a simple taxonomic key including the newly described species and the other *Leucoagaricus* and *Leucocoprinus* species from China.

### ﻿An artificial key to the species of *Leucoagaricus* reported from China

**Table:** 

1	Pileus yellow, with golden droplets exuding	***La. exannulatus***
–	Pileus without droplets exuding	**2**
2	Annulus with orange or pink-purplish distinct rim	***La. lateritiopurpureus***
–	Annulus white or not white	**3**
3	Pileus central dark brown to blackish brown	**4**
–	Pileus central pale yellow, orange, red, brown, or concolorous margin	**5**
4	Pileus with depressed dark brownish to grayish brown squamules; stipe covered with purplish brown flocculent fibers; annulus easily detachable	***La. cinereibisporus***
–	Pileus covered with blackish brown to fuliginous warted or felted squamules; stipe nearly glabrous; annulus white, fugacious	***La. atrosquamulosus***
5	Pileus smooth to glabrous, occasionally with radial fibrils	***La. subnivalis***
–	Pileus with squamules or fibrils	**6**
6	Pileus with radial streaks towards the margin or fragile	**7**
–	Pile margin entire edges without streaks	**8**
7	Pileus centre yellowish orange, margin with radial streaks; basidia clavate	***La. centrobrunneolus***
–	Pileus center slightly yellowish, margin ruptured and fragile; cheilocystidia narrowly clavate	***La. subcandidus***
8	Stipe enlarged to subclavate towards base and ranges from cylindrical	**9**
–	Stipe cylindrical or slightly expanded at the base	**10**
9	Pileus covered with orange-brown or red-brown floccose squamules; annulus with serrulate margin	***La. bulbosus***
–	Pileus covered with radially arranged light brownish orange to yellowish-orange fibrils or squamules	***La. xantholepis***
10	Pileus umbo becoming radially fissured from the center upon maturation, covered with imbricate squamules and adiating fibrillose vestiges	***La. bailangshanensis***
–	Pileus umbo entire, not issured	**11**
11	Pileus reddish brown to yellowish brown; cheilocystidia narrowly clavate, often wavy in shape	***La. testaceumbonatus***
–	Pileus centre yellowish to light brownish, elsewhere with radially arranged light brownish squamules; cheilocystidia apex often sub-capitate to capitate	***La. luteocanus***

### ﻿An artificial key to the species of *Leucocoprinus* reported from China

**Table:** 

1	Basidiocarps small	**2**
–	Basidiocarps large	**18**
2	Pileus or context discoloring when bruised	**3**
–	Pileus or context not discoloring when bruised	**4**
3	Pileus discoloring dark green when bruised	***Lc. virens***
–	Pileus discoloring oxide red when bruised	***Lc. ferrugineus***
4	Stipe bulbous at base	***Lc. bulbiger***
–	Stipe not bulbous at base	**5**
5	Pileus center dark reddish brown	***Lc. candidus***
–	Pileus center not dark reddish brown	**6**
6	Cheilocystidia with refractive contents	***Lc. aurantioruber***
–	Cheilocystidia without refractive contents	7
7	Pileus purplish	**8**
–	Pileus not purplish	**10**
8	Context discoloring purplish when drying	***Lc. purpurascens***
–	Context not discoloring when drying	**9**
9	Pileus with dark brown umbo; stipe base faint lilacinous tinge.	***Lc. purpureolilacinus***
–	Pileus with dark umbo; spores with germ pore	***Lc. brebissonii***
10	Cheilocystidiapyriform; lamellae margin light brownish	***Lc. beijingensis***
–	Cheilocystidia not pyriform	**11**
11	Pileus with farinaceous veil	***Lc. griseofloccosus***
–	Pileus without veil	**12**
12	Pileus margin ruptured; covered with light brownish scales	***Lc. digitatocystis***
–	Pileus margin not ruptured	**13**
13	Cheilocystidia have refractive contents at the apex	**14**
–	Cheilocystidia without refractive contents at the apex	**15**
14	Pileus with green-gray scales; cheilocystidia apex with crystals	***Lc. subcrystallifer***
–	Pileus with orange brown to red brown floccose scales	***Lc. centricastaneus***
15	Spores ovoid	**16**
–	Spores not ovoid	**17**
16	Pileus white, margin incurved	***Lc. pakistaniensis***
–	Pileus with dark reddish-brown scales	***Lc. furfuraceipes***
17	Annulus margin brownish black to black	***Lc. brunneocanus***
–	Annulus white, pinkish to pinkish-tan when aging	***Lc. leucothites***
18	Pileus bright yellow	***Lc. birnbaumii***
–	Pileus not bright yellow	**19**
19	Pileus truncated campanulate	***Lc. truncatus***
–	Pileus not truncated campanulate	**20**
20	Basidia spheropedunculate; cheilocystidiasubfusiform and mucronate	***Lc. cretaceus***
–	Basidia not spheropedunculate	**21**
21	Pileus center dark brown; pileipellis with yellow hyphae	***Lc. subpurpureolilacinus***
–	Pileus center not dark brown	**22**
22	Pileus smooth; annulus yellowish white	***Lc. orientiflavus***
–	Pileus without adnate scales	**23**
23	Annulus peronate	***Lc. rubrotinctus***
–	Annulus not peronate	**24**
24	Pileus very fragile, grooved from the margin to the center	***Lc. fragilissimus***
–	Pileus not very fragile	**25**
25	Cheilocystidia apex with tiny crystals	***Lc. croceovelutinus***
–	Cheilocystidia apex without tiny crystals	**26**
26	Pileus with golden yellow, reddish brown watery exudates	***Lc. lacrymans***
–	Pileus without watery exudates	**27**
27	Pileus with dirty brown scales; stipe base reddish brown	***Lc. subglobisporus***
–	Pileus without dirty brown scales	**28**
28	Cheilocystidia narrowly clavate	***Lc. tangerinus***
–	Cheilocystidia not narrowly clavate	**29**
29	Lamellae pale yellow, cheilocystidia lageniform	***Lc. straminellus***
–	Lamellae not pale yellow	**30**
30	Pileus with green radially scales; cheilocystidia with crystals inside	***Lc. atroviridis***
–	Pileus scales not green	**31**
31	Stipe with pale brown scales below anulus	***Lc. shixingensis***
–	Stipe without scales below anulus	**32**
32	Cheilocystidia fusiform	***Lc. cygneus***
–	Cheilocystidia ventricose, fusiform, lageniform, and some rostrate	***Lc. cepistipes***

## Supplementary Material

XML Treatment for Leucoagaricus bailangshanensis

XML Treatment for Leucocoprinus ferrugineus

## ﻿Additional information

### Use of AI

No use of AI was reported.

### Author ORCIDs

Entaj Tarafder https://orcid.org/0000-0002-3680-3433

Ji-Ze Xu https://orcid.org/0000-0003-2082-8529

### Data availability

All of the data that support the findings of this study are available in the main text.
